# Effectiveness of overground robotic exoskeletons in early inpatient rehabilitation for incomplete spinal cord injury: a systematic review protocol

**DOI:** 10.3389/fnins.2026.1781656

**Published:** 2026-03-10

**Authors:** Ravi Shankar, Nur Shafawati, Gobinathan Chandran

**Affiliations:** 1 Clinical Research & Innovation Office, Tan Tock Seng Hospital, National Healthcare Group, Singapore, Singapore; 2 Department of Rehabilitation, Alexandra Hospital, National University Health System, Singapore, Singapore; 3 Division of Rehabilitation Medicine, Department of Medicine, National University Hospital, National University Health System, Singapore, Singapore; 4 Yong Loo Lin School of Medicine, National University of Singapore, Singapore, Singapore

**Keywords:** gait rehabilitation, incomplete spinal cord injury, inpatient rehabilitation, neuroplasticity, robotic exoskeleton, spinal cord injury

## Abstract

**Background:**

Spinal cord injury affects approximately 9 million people worldwide, with incomplete injuries representing the majority of cases and offering greater potential for functional recovery. Overground robotic exoskeletons have emerged as promising rehabilitation tools, yet evidence regarding their effectiveness specifically during early inpatient rehabilitation remains inadequately synthesised.

**Objectives:**

To systematically evaluate the effectiveness of overground robotic exoskeletons compared to conventional rehabilitation for improving walking function in adults with incomplete spinal cord injury during early inpatient rehabilitation.

**Methods:**

This protocol follows PRISMA-P guidelines. Searches will be conducted across MEDLINE, Embase, CINAHL, Cochrane Library, and PEDro from inception through December 2025. Randomised and non-randomised controlled trials will be included. Two reviewers will independently screen studies using Covidence, extract data, and assess risk of bias using RoB 2 and ROBINS-I tools. Meta-analysis will be conducted where appropriate.

**Discussion:**

This protocol establishes a rigorous framework for synthesising evidence on early exoskeleton-assisted rehabilitation, addressing a critical gap in clinical practice guidelines for spinal cord injury rehabilitation.

## Introduction

Spinal cord injury represents a devastating neurological condition with profound implications for individuals, healthcare systems, and society. The Global Burden of Disease Study 2019 estimated approximately 9 million prevalent cases of spinal cord injury worldwide, representing a 52.7% increase compared to 1990 (Ding et al., 2022; Liu et al., 2023). A comprehensive meta-analysis of 229 studies reported an overall incidence rate of 23.77 per million population, with traumatic spinal cord injury occurring at 26.48 per million (Lu et al., 2024). The economic burden is substantial, with annual direct costs exceeding $9.7 billion in the United States and lifetime treatment costs per patient ranging from $1.1 to $4.7 million depending on injury severity (Ding et al., 2022). These figures underscore the critical importance of optimising rehabilitation interventions to maximise functional recovery and reduce long-term disability.

Spinal cord injuries are classified according to the American Spinal Injury Association Impairment Scale (AIS), which distinguishes complete injuries (AIS A) from incomplete injuries (AIS B, C, D) based on preserved sensorimotor function below the neurological level of injury (Roberts et al., 2017). Incomplete injuries, which constitute approximately 60–70% of traumatic spinal cord injuries, retain some neural pathways and consequently offer substantially greater potential for functional recovery through neuroplasticity mechanisms (Kirshblum et al., 2021). Individuals classified as AIS C or D, who retain some motor function, demonstrate particularly favourable prognosis for walking recovery, with studies indicating that 75% of AIS C patients and nearly all AIS D patients achieve some degree of functional ambulation within the first year post-injury (van Middendorp et al., 2009; Burns et al., 2011).

The focus on incomplete injuries is further justified by the mechanism of action of overground robotic exoskeletons, which require residual voluntary motor activity to provide augmented sensorimotor feedback and facilitate activity-dependent neuroplasticity. Individuals with incomplete injuries retain descending neural pathways that can be engaged through repetitive exoskeleton-assisted gait training, making this population the most physiologically appropriate target. Additionally, AIS C and D injuries represent the group for whom clinical equipoise exists regarding whether exoskeleton training provides benefit beyond conventional intensive physiotherapy.

The timing of rehabilitation initiation is increasingly recognised as a critical factor influencing outcomes. Early intensive rehabilitation, initiated within the acute and subacute phases following injury, capitalises on heightened neural plasticity during this period and may establish motor patterns before maladaptive compensatory strategies become entrenched (Dobkin et al., 2003). The inpatient rehabilitation phase, typically occurring within the first three months post-injury, represents a crucial therapeutic window during which the most rapid neurological recovery occurs and foundational mobility skills are established (Kirshblum et al., 2021). Despite this recognition, optimal rehabilitation protocols during this early phase remain incompletely defined, with substantial variation in clinical practice across centres and healthcare systems.

Robotic exoskeletons have emerged as innovative rehabilitation tools offering the potential for intensive, repetitive, and standardised gait training that may complement or enhance conventional physiotherapy approaches. These devices are broadly categorised into treadmill-based systems such as Lokomat and overground exoskeletons including Ekso, ReWalk, Indego, and HAL (He et al., 2024). Overground exoskeletons enable walking in real-world environments, providing ecological validity and enhanced visual, vestibular, and proprioceptive feedback that may promote motor learning and neuroplasticity (Mekki et al., 2018). Recent meta-analyses have demonstrated that robotic exoskeleton gait training can significantly improve walking outcomes in spinal cord injury populations, with improvements observed in walking speed, endurance, balance, and functional independence measures (Guo et al., 2025; Moriarty et al., 2024; Liu et al., 2025).

However, the existing evidence base presents important limitations for clinical translation. A recent scoping review identified that most exoskeleton research has been conducted in chronic injury populations, with only a minority of studies examining outcomes during early rehabilitation phases (Miller et al., 2016). Furthermore, systematic reviews have typically combined heterogeneous populations including both complete and incomplete injuries, treadmill-based and overground devices, and acute through chronic injury phases, potentially obscuring treatment effects specific to clinical subgroups (Mehrholz et al., 2017). A dose–response analysis of exoskeleton rehabilitation parameters noted substantial protocol variability across studies, highlighting the need for synthesis focused on specific clinical contexts (Nepomuceno et al., 2024). This planned systematic review addresses these gaps by focusing specifically on overground exoskeletons in the early inpatient rehabilitation phase for individuals with incomplete spinal cord injury.

The primary objective of this planned systematic review is to evaluate the effectiveness of overground robotic exoskeleton-assisted gait training compared to conventional rehabilitation for improving walking function in adults with incomplete spinal cord injury during early inpatient rehabilitation. Effectiveness will be assessed through examination of walking speed, walking endurance, functional mobility, and walking independence as measured by validated outcome instruments.

Secondary objectives are organised into two categories. Clinical outcome objectives: evaluate the effects of overground exoskeleton training on (a) lower extremity motor strength (LEMS), (b) balance (Berg Balance Scale, Timed Up and Go), (c) functional independence (SCIM-III), and (d) spasticity (Modified Ashworth Scale). Evidence mapping objectives: (a) characterise dose and dosage parameters (session duration, frequency, total sessions, intensity progression), (b) examine adverse events and safety profiles, (c) explore heterogeneity in treatment effects across injury severity, neurological level, time since injury, and intervention parameters, and (d) identify evidence gaps for future trial design.

The review question follows the PICO framework as recommended for intervention reviews (Chandler et al., 2019). The population comprises adults with incomplete traumatic or non-traumatic spinal cord injury classified as AIS B, C, or D receiving inpatient rehabilitation within six months of injury onset. The intervention is overground robotic exoskeleton-assisted gait training using powered lower limb exoskeletons. The comparator is conventional rehabilitation including standard physiotherapy, body-weight supported treadmill training, or other non-exoskeleton gait training approaches. Primary outcomes include walking speed measured by the 10-metre walk test, walking endurance measured by the 6-min walk test, and functional walking ability measured by the Walking Index for Spinal Cord Injury II (WISCI-II).

## Methods

### Study design and registration

This protocol was developed in accordance with the Preferred Reporting Items for Systematic Review and Meta-Analysis Protocols (PRISMA-P) statement (Shamseer et al., 2015). The completed review will be reported following PRISMA 2020 guidelines (Page et al., 2021). The protocol will be prospectively registered with PROSPERO prior to initiating the literature search to ensure transparency and reduce selective reporting bias. Methodological conduct will follow the Cochrane Handbook for Systematic Reviews of Interventions (Chandler et al., 2019) and established guidance for systematic reviews of rehabilitation interventions.

### Eligibility criteria

Studies will be selected according to predefined eligibility criteria relating to population, intervention, comparator, outcomes, and study design. The criteria were developed to ensure the review captures evidence directly relevant to clinical decision-making regarding early exoskeleton rehabilitation while maintaining sufficient sensitivity to identify all potentially relevant studies. The Table 1 summarises inclusion and exclusion criteria.

**Table 1:** Inclusion and exclusion criteria for study selection.

Criterion	Inclusion	Exclusion
Population	Adults (≥18 years) with incomplete SCI (AIS B, C, or D); traumatic or non-traumatic aetiology; within 6 months of injury onset; receiving inpatient rehabilitation	Complete SCI (AIS A); chronic injury (>6 months post-onset); outpatient or community settings; paediatric populations; congenital conditions
Intervention	Overground powered robotic exoskeletons (e.g., Ekso, ReWalk, Indego, HAL, ABLE Exoskeleton) providing bilateral hip and knee actuation for gait training	Treadmill-based robotic devices (e.g., Lokomat); passive orthoses; single-joint devices; upper limb exoskeletons; functional electrical stimulation alone
Comparator	Conventional physiotherapy; body-weight supported treadmill training; overground gait training without exoskeleton; standard inpatient rehabilitation	No exclusions based on comparator type
Outcomes	Primary: 10MWT, 6MWT, WISCI-II; Secondary: LEMS, TUG, Berg Balance Scale, SCIM-II, Modified Ashworth Scale	Studies not reporting at least one walking-related outcome
Study design	Randomised controlled trials; quasi-randomised trials; non-randomised controlled trials with concurrent comparison groups	Single-arm studies; case series; case reports; before-after studies without control; cross-sectional studies; qualitative studies
Publication	Peer-reviewed journal articles; conference papers with full methodology; any language; database inception through December 2025	Abstracts without full text; editorials; protocols without results; duplicate publications

### Comparator classification framework

Given heterogeneity of control conditions in rehabilitation trials, comparator interventions will be classified into four categories:

Category 1, Conventional physiotherapy: Standard physiotherapy-led gait training including manual-assisted overground walking, standing frame activities, parallel bar training, and lower limb strengthening without robotic or treadmill components.
Category 2, Body-weight supported treadmill training (BWSTT): Treadmill-based gait training with partial body-weight support via overhead harness, with or without manual therapist assistance.
Category 3, Overground gait training without exoskeleton: Structured overground walking using conventional assistive devices (walkers, crutches, orthoses) without powered robotic assistance.
Category 4, Standard inpatient rehabilitation (multicomponent): Usual care programmes combining multiple modalities where the specific gait training component cannot be isolated.

Classification will be performed independently by two reviewers. Where descriptions are insufficient, study authors will be contacted. This framework will inform subgroup and sensitivity analyses.

### Information sources and search strategy

A comprehensive search strategy will be implemented across multiple electronic databases to identify all potentially relevant studies. Primary databases include MEDLINE via PubMed, Embase via Elsevier, CINAHL via EBSCO, and the Cochrane Central Register of Controlled Trials. Specialist rehabilitation databases including PEDro (Physiotherapy Evidence Database) and REHABDATA will be searched. Trial registries including ClinicalTrials.gov and the WHO International Clinical Trials Registry Platform will be searched to identify ongoing or completed trials with unpublished results. Reference lists of included studies and relevant systematic reviews will be hand-searched, with forward citation tracking via Google Scholar to identify citing articles.

The search strategy was developed with input from an information specialist with expertise in rehabilitation systematic reviews. Search terms combine controlled vocabulary (MeSH, Emtree) with free-text terms addressing the population (spinal cord injury, paraplegia, tetraplegia), intervention (exoskeleton, robotic, powered orthosis, wearable robot), and study design (randomised, controlled trial). No methodological filters will be applied to maximise sensitivity. Searches will cover database inception through December 2025, with no language restrictions. Non-English articles will be translated using professional services where necessary.

The following example search string will be used for PubMed/MEDLINE:

("Spinal Cord Injuries"[MeSH] OR spinal cord injur*[tiab] OR SCI[tiab] OR paraplegia[tiab] OR tetraplegia[tiab] OR quadriplegia[tiab] OR "Paraplegia"[MeSH] OR "Quadriplegia"[MeSH]) AND ("Exoskeleton Device"[MeSH] OR exoskeleton*[tiab] OR "powered orthosis"[tiab] OR "robotic gait"[tiab] OR "robot assisted gait"[tiab] OR "wearable robot*"[tiab] OR ReWalk[tiab] OR Ekso[tiab] OR Indego[tiab] OR HAL[tiab] OR "hybrid assistive limb"[tiab] OR ABLE[tiab]) AND ("Gait"[MeSH] OR "Walking"[MeSH] OR gait[tiab] OR walk*[tiab] OR locomot*[tiab] OR ambula*[tiab] OR "Rehabilitation"[MeSH] OR rehabilitat*[tiab])

The strategy will be adapted for each database according to specific syntax requirements, with complete documentation provided in Supplementary materials.

### Study selection

Study selection will be conducted using Covidence systematic review software (Veritas Health Innovation, Melbourne, Australia), enabling independent duplicate screening with automated conflict detection. The selection process will proceed in two stages. In the first stage, two reviewers will independently screen titles and abstracts against eligibility criteria, with potentially relevant records proceeding to full-text review. In the second stage, full texts will be retrieved and independently assessed by two reviewers, with exclusion reasons documented according to a predefined hierarchy. Disagreements will be resolved through discussion and, where necessary, consultation with a third reviewer with expertise in spinal cord injury rehabilitation. The screening process will be piloted on 50 records to calibrate reviewer judgements and refine criteria definitions. Inter-rater reliability will be calculated using Cohen’s kappa statistic. A PRISMA 2020 flow diagram will document the selection process (Page et al., 2021).

### Data extraction

Data will be extracted using a standardised form developed in Covidence and pilot-tested on three included studies. Extracted information will encompass study characteristics (authors, year, country, design, setting, funding); participant characteristics (sample size, age, sex, injury aetiology, neurological level, AIS grade, time since injury); intervention details (exoskeleton device, manufacturer, training protocol, session duration, frequency, total sessions, total training time, assistance level, progression criteria); comparator details (type, intensity, duration); and outcome data (outcome measures, assessment timepoints, mean values, standard deviations, sample sizes by group). Comparator interventions will be characterised per the predefined classification framework (Categories 1 to 4), with extraction of session duration, frequency, total sessions, use of body-weight support (yes/no, percentage), level of therapist assistance, and co-interventions. For continuous outcomes, means and standard deviations will be extracted or calculated from reported statistics. Where necessary, graphical data will be extracted using validated software (WebPlotDigitizer). Two reviewers will independently extract data, with discrepancies resolved through consensus. Study authors will be contacted for missing data, with a maximum of three contact attempts over six weeks.

### Quality assessment

Methodological quality of included randomised controlled trials will be assessed using the Cochrane Risk of Bias 2 (RoB 2) tool, which evaluates bias across five domains: randomisation process, deviations from intended interventions, missing outcome data, measurement of the outcome, and selection of reported results (Sterne et al., 2019). For non-randomised controlled trials, the Risk of Bias in Non-randomised Studies of Interventions (ROBINS-I) tool will be employed, assessing bias due to confounding, selection, classification of interventions, deviations, missing data, outcome measurement, and selection of reported results (Sterne et al., 2016). Each domain will be judged as low risk, some concerns, or high risk for RoB 2, or low, moderate, serious, critical, or no information for ROBINS-I. An overall risk of bias judgement will be assigned to each study. Quality assessment will be performed independently by two reviewers, with disagreements resolved through discussion. Results will be presented using Cochrane risk of bias summary figures and will inform sensitivity analyses and GRADE certainty assessments.

### Risk of bias assessment

Beyond individual study quality, sources of bias at the review level will be assessed. Publication bias will be evaluated using funnel plot asymmetry where at least ten studies report a given outcome, supplemented by Egger’s regression test (Egger et al., 1997). Selective outcome reporting will be assessed by comparing protocols or trial registrations with published reports where available. Small-study effects will be examined through sensitivity analyses excluding studies with sample sizes below the median. The potential impact of missing data will be explored through best-case and worst-case scenario analyses. Conflicts of interest and funding sources will be documented and considered when interpreting findings, as industry sponsorship has been identified as a potential source of bias in rehabilitation technology research (Charette et al., 2023).

### Data synthesis

Meta-analysis will be conducted where at least three studies report comparable outcomes using similar measurement instruments in sufficiently homogeneous populations. For continuous outcomes, standardised mean differences (SMD) with 95% confidence intervals will be calculated using Hedges’ g to correct for small sample bias. Where studies use identical outcome measures, mean differences (MD) will be calculated to facilitate clinical interpretation. Random effects models using the restricted maximum likelihood estimator will be employed to account for anticipated between-study heterogeneity. Statistical heterogeneity will be quantified using the I^2^ statistic, with values exceeding 50% indicating substantial heterogeneity warranting exploration (Higgins et al., 2003). Prediction intervals will be calculated to indicate the range of treatment effects expected in future settings.

Heterogeneity will be explored through pre-specified subgroup analyses examining injury severity (AIS B versus C versus D), neurological level (cervical versus thoracic versus lumbar), time since injury (acute <1 month versus subacute 1–6 months), intervention intensity (total training hours), and exoskeleton device type. Additional subgroup analyses will examine treatment effects by comparator type (Categories 1 to 4) to determine whether exoskeleton benefit varies by the nature and intensity of the control intervention. Sensitivity analyses restricted to active comparators (Categories 1 to 3) will assess robustness of effects compared to structured gait training rather than general standard care, addressing the potential for inflated effect sizes when exoskeletons are compared against less intensive protocols. Meta-regression will be considered where at least ten studies are available. Sensitivity analyses will examine robustness of findings to exclusion of high risk of bias studies, non-randomised studies, and studies with imputed data. Where meta-analysis is inappropriate due to clinical or methodological heterogeneity, narrative synthesis following SWiM guidance will be employed (Campbell et al., 2020). The certainty of evidence for each outcome will be assessed using the GRADE approach, considering risk of bias, inconsistency, indirectness, imprecision, and publication bias (Guyatt et al., 2008). Analyses will be conducted using Review Manager 5.4 and R statistical software.

## Discussion

This protocol establishes the methodological framework for a systematic review addressing a critical evidence gap in spinal cord injury rehabilitation. While recent meta-analyses have demonstrated overall benefits of robotic exoskeleton training for walking outcomes in spinal cord injury populations (Guo et al., 2025), no synthesis has focused specifically on the early inpatient rehabilitation phase for incomplete injuries. This phase represents a crucial therapeutic window when neurological recovery potential is maximal and foundational mobility skills are established, making the evidence regarding optimal rehabilitation approaches during this period of particular clinical importance.

The anticipated findings of this planned review will have significant implications for clinical practice and policy. Rehabilitation physicians, physiotherapists, and multidisciplinary teams will benefit from synthesised evidence to guide decisions about incorporating overground exoskeletons into early inpatient rehabilitation programmes. Current clinical practice guidelines, including those from the Paralyzed Veterans of America and international consensus statements, acknowledge the potential role of robotic technologies but note insufficient evidence to make specific recommendations regarding timing, patient selection, and dosing parameters (Kirshblum et al., 2021). This review will provide the evidence foundation necessary to inform such recommendations. Health technology assessment bodies and healthcare administrators will gain insight into the comparative effectiveness of exoskeleton technologies, supporting resource allocation decisions in an era of constrained healthcare budgets.

Individuals with spinal cord injury and their families will benefit from improved access to evidence-based information regarding rehabilitation options. Given that walking recovery is consistently identified as a top priority by individuals with incomplete spinal cord injury (Anderson, 2004), clear evidence regarding interventions that may enhance walking outcomes is of paramount importance to this community. Researchers will gain understanding of existing evidence gaps and methodological limitations, informing the design of future trials. The characterisation of intervention protocols and dose–response relationships will provide essential information for designing studies with optimal training parameters.

Several methodological considerations warrant discussion at the protocol stage. First, the focus on incomplete injuries (AIS B, C, D) reflects the greater recovery potential, mechanistic alignment between residual neural pathways and exoskeleton-mediated sensorimotor feedback, and the need to reduce heterogeneity that has limited interpretability of previous reviews combining complete and incomplete injuries (Mehrholz et al., 2017). We acknowledge this limits generalisability to individuals with complete injuries (AIS A), for whom exoskeletons may serve different goals such as cardiovascular conditioning and psychological wellbeing rather than functional ambulation recovery. This may also reduce eligible studies, limiting precision of effect estimates. To mitigate this, we will report the number and characteristics of excluded studies involving complete or mixed populations to contextualise the evidence base and inform future reviews with broader criteria. Second, the restriction to early inpatient rehabilitation within six months of injury onset ensures relevance to clinical decision-making during this critical therapeutic window but excludes chronic populations where much exoskeleton research exists. This precludes assessment of long-term outcome durability. Where included studies report follow-up assessments beyond the intervention period (e.g., at discharge or 3 to 6 month follow-up), these data will be extracted to provide preliminary insights into sustainability of treatment effects. The absence of long-term data will be explicitly identified as an evidence gap, with recommendations for future trials to incorporate extended follow-up to evaluate whether early exoskeleton gains translate into lasting improvements in community settings. Third, distinguishing overground from treadmill-based devices reflects important differences in ecological validity, motor learning demands, and practical implementation, but some studies may combine or compare these approaches in ways that complicate categorisation.

Anticipated challenges include heterogeneity of exoskeleton devices, training protocols, and outcome measures. Overground exoskeletons vary in actuation mechanisms, control strategies (pre-programmed vs. user-intent-based), and adjustable assistance levels, which may differentially influence motor learning outcomes. A recent dose–response analysis identified substantial variability in session parameters across studies (Nepomuceno et al., 2024). To address this: (a) detailed device technical specifications and control approaches will be extracted and tabulated, (b) subgroup analyses by device type will be conducted where sufficient studies exist, (c) outcome measure heterogeneity will be managed by prioritising validated instruments (10MWT, 6MWT, WISCI-II) as primary outcomes and using standardised mean differences for secondary outcomes, and (d) where pooling is inappropriate (I^2^ > 75%), narrative synthesis following SWiM guidance will be used with transparent reporting of heterogeneity sources. Despite these strategies, device variability may limit specificity of recommendations for individual platforms. Publication bias may affect the evidence base, as positive findings may be more likely to be published, particularly for studies supported by device manufacturers. The comprehensive search strategy including trial registries and manufacturer-independent databases aims to mitigate this risk.

The planned review will make several important contributions regardless of specific findings. It will provide the first comprehensive mapping of controlled studies evaluating overground exoskeletons specifically in early inpatient rehabilitation for incomplete spinal cord injury. The synthesis will characterise intervention protocols and dose–response relationships, informing both clinical practice and future research design. Critical appraisal of methodological quality will highlight strengths and limitations affecting evidence reliability. Identification of evidence gaps will guide prioritisation of future research. Finally, the review will produce a resource that can inform clinical practice guidelines, health technology assessment, and shared decision-making between clinicians and patients regarding early rehabilitation approaches.

In conclusion, this protocol presents a rigorous methodological framework for systematically reviewing the effectiveness of overground robotic exoskeletons in early inpatient rehabilitation for incomplete spinal cord injury. The focus on this specific population, intervention type, and rehabilitation phase addresses limitations of previous syntheses and will provide evidence directly applicable to clinical decision-making. The comprehensive search strategy, transparent screening procedures using Covidence, validated quality assessment tools, and appropriate synthesis methods ensure a high-quality review that can meaningfully inform policy and practice in this important area of neurological rehabilitation.
